# Efavirenz use and contraceptive methods in HIV-positive women in a large urban cohort

**DOI:** 10.1186/1758-2652-13-S4-P112

**Published:** 2010-11-08

**Authors:** RA Seaton, R Fox, A MacConnachie, P Anderson, R Nandwani, E Peters, A Winter, R Taylor

**Affiliations:** 1 Gartnavel General, Infection Unit, Brownlee Centre, Glasgow, UK; 2 Sandyford Initiative, Glasgow, United Kingdom; 3 Sandyford, Genitourinary Medicine, Glasgow, UK

## Background

Despite increasing reports of successful pregnancies whilst using Efavirenz (EFZ), the drug remains Category C during pregnancy due to concerns around teratogenicity [1]. Additionally, EFZ can render many hormonal methods of contraception less effective. For these reasons, UK guidance suggests that HIV positive women should be informed of these effects before commencing treatment [2]. Following a case in this unit where a young HIV-positive woman had an unplanned pregnancy whilst using Implanon and taking EFZ/Truvada, we examined contraceptive use and advice given to women in our cohort using EFZ, and then instigated changes to improve practice in this area.

## Methods

Case-note review of all women taking EFZ in Jan 2008 and again in Feb 2010. Current contraception used, advice on teratogenicity, and advice on efficacy documentation was recorded. Women over 50, with documented menopause or hysterectomy were excluded.

## Results

**Figure 1:**
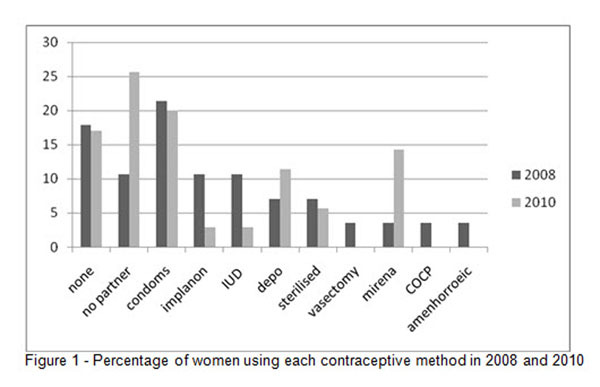


In 2008 we identified 31 females using EFZ in our cohort of 912 patients. Contraceptive choices are shown in Figure 1. 68% were using an 'effective' method of contraception (one not liable to reduced efficacy when using EFZ - condoms, IUS/IUD, sterilisation or recently documented no partner). 36% had documented advice regarding teratogenicity and 75% regarding reduced efficacy of hormonal methods. Following these results we introduced a section for contraception on our clinical review form (which is updated at each HIV clinic review) to act as a prompt for clinicians. After this change was made, we re-examining these data following this in 2010 (See Fig 1) and found 35 females using EFZ. 80% were using an 'effective' method of contraception, 50% had documented advice on teratogenicity and 100% regarding reduced efficacy of hormonal contraception (if appropriate).

## Conclusions

Simple changes such as adding contraception to a clinic proforma can help improve sexual and reproductive health outcomes in HIV positive women. However, there are still improvements to be made in documentation of advice given, particularly when using a Category C drug in women who may become pregnant. Additionally, women should be made aware of the potential interaction between antiretrovirals and hormonal contraceptives at the HIV clinic — particularly as some may not disclose their status to Family Planning or GP services and therefore we cannot assume that this advice is being given elsewhere.
